# Initial experience of enfortumab vedotin in a patient with metastatic urothelial carcinoma on hemodialysis: Two case reports

**DOI:** 10.1002/iju5.12736

**Published:** 2024-05-26

**Authors:** Shintaro Mori, Tomohiro Matsuo, Hiroyuki Honda, Kyohei Araki, Kensuke Mitsunari, Kojiro Ohba, Yasushi Mochizuki, Ryoichi Imamura

**Affiliations:** ^1^ Department of Urology Nagasaki University Graduate School of Biomedical Sciences Nagasaki Japan

**Keywords:** end‐stage renal disease, enfortumab vedotin, hemodialysis, metastatic urothelial carcinoma

## Abstract

**Introduction:**

Few studies have reported on administering enfortumab vedotin to patients with metastatic urothelial carcinoma and end‐stage renal disease requiring hemodialysis.

**Case presentation:**

Case 1: An 85‐year‐old man underwent hemodialysis for progressive renal failure 4 months after right laparoscopic radical nephroureterectomy. Case 2: A 73‐year‐old man underwent hemodialysis after two laparoscopic radical nephroureterectomies for recurrent urothelial carcinoma. In both cases, enfortumab vedotin was administered due to postoperative recurrence and progression despite platinum‐based chemotherapy and pembrolizumab. Partial response and disease progression were observed in cases 1 and 2, respectively. Adverse events included a mild skin rash in both patients and neutropenia in Case 1, both of which resolved with symptomatic treatment.

**Conclusion:**

The efficacy and safety of enfortumab vedotin in patients with metastatic urothelial carcinoma, and end‐stage renal disease undergoing hemodialysis, were confirmed.

**Keynote message**

We report that EV administration in two patients with metastatic urothelial carcinoma undergoing hemodialysis was safe, while showing some efficacy.

**Abbreviations & Acronyms**
AE: adverse event
BCG: Bacille Calmette‐Guérin
BSC: best supportive care
BV: brentuximab vedotin
CT: computed tomography
ESRD: end‐stage renal disease
EV: enfortumab vedotin
ICI: immune checkpoint inhibitor
MMAE: monomethyl auristatin E
mUC: metastatic urothelial carcinoma
PD: progression of disease
PR: partial response
RECIST: response evaluation criteria in solid tumors
TURBT: transurethral resection of a bladder tumor
UTUC: upper tract urothelial carcinoma

## Introduction

The treatment of mUC is challenging while anticipating the development of new therapeutic agents. EV is now used in several countries, including Japan, for the treatment of mUC that progresses after chemotherapy or ICIs treatment. Despite supporting evidence for EV's efficacy and safety, patients with mUC and ESRD on hemodialysis were not included, even in large‐scale clinical studies.^1^, ^2^ There have been only a few reports on the administration of EV in patients undergoing hemodialysis for mUC. Hence, discussing their efficacy and safety in real‐world situations is important. This report presents cases of two patients undergoing hemodialysis who received EV for mUC.

## Case presentation

### Patient 1

**Fig. 1:**
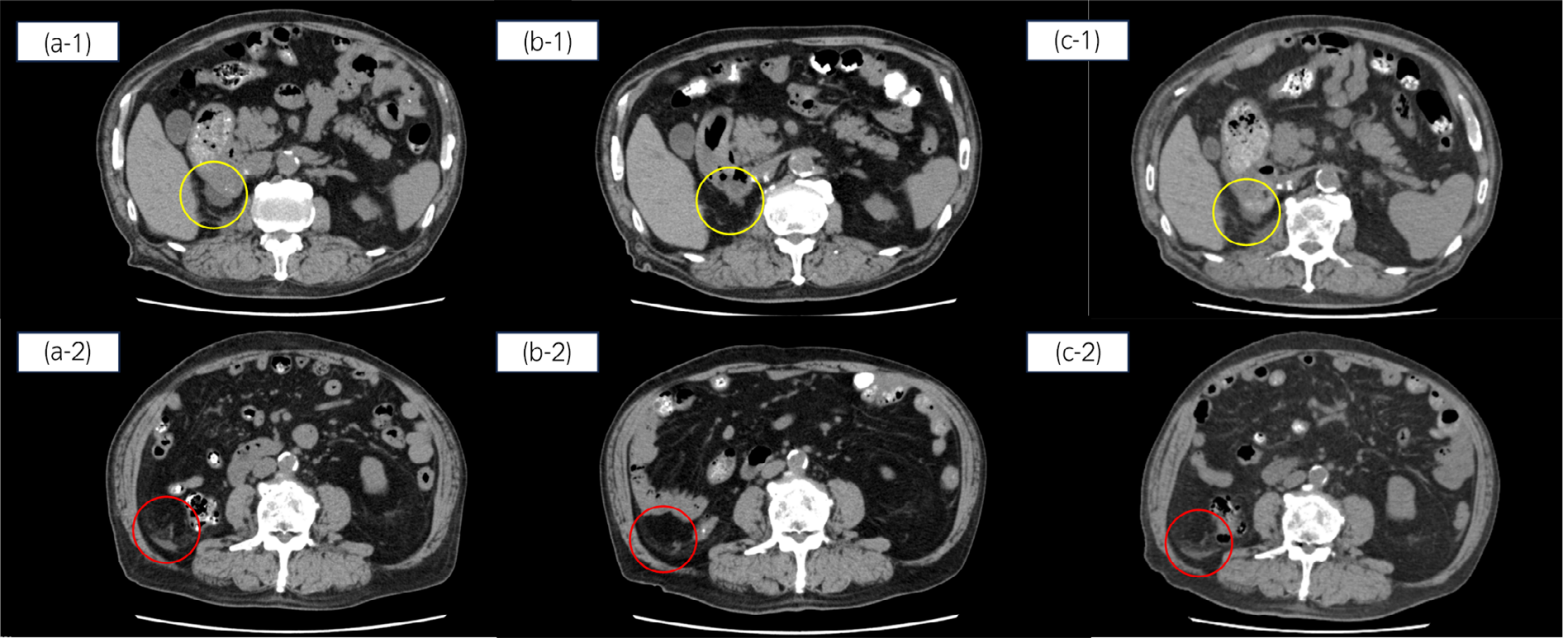
CT findings in Patient 1. CT from February X before EV treatment demonstrating disseminated two lesions of the peritoneum (yellow and red circles, a‐1, ‐2), from April X after two cycles of EV demonstrating PR of both lesions (b‐1, ‐2), and from July X after four cycles of EV demonstrating PD, which is an increase in the size of the both lesions (c‐1, ‐2).

**Fig. 2:**
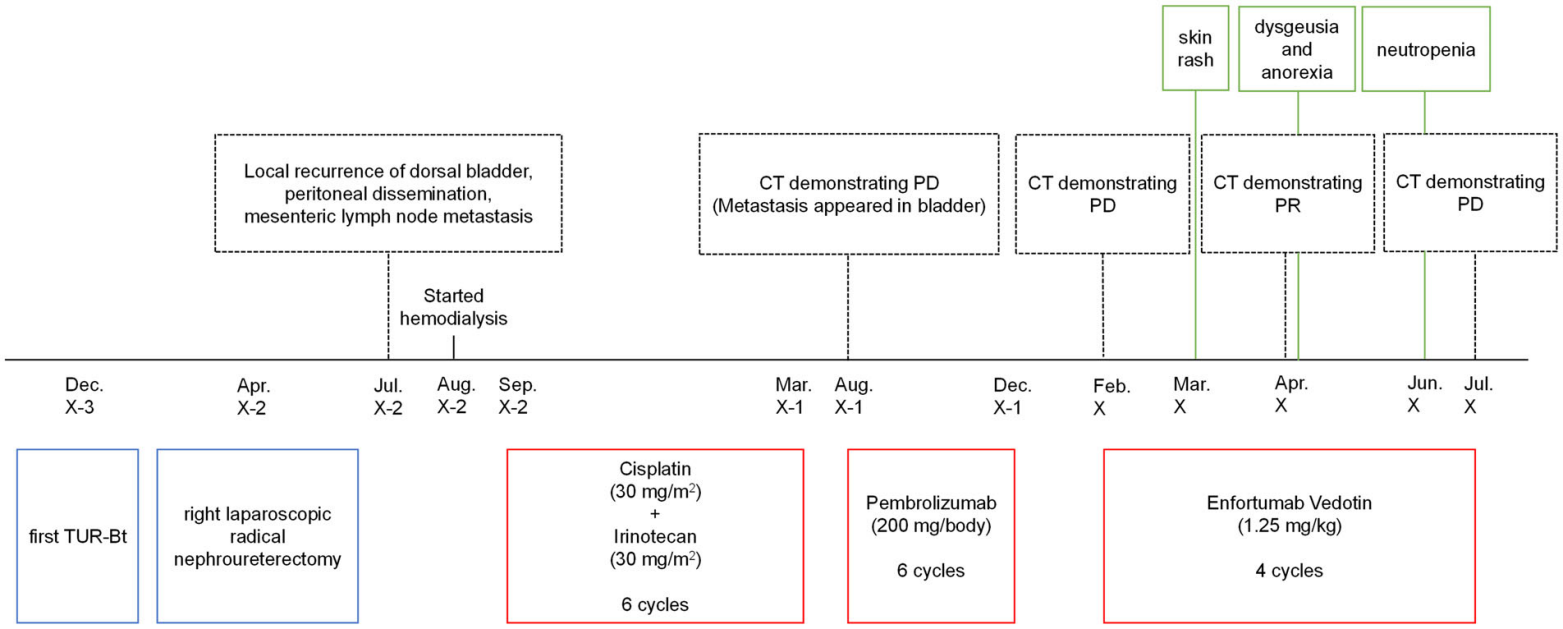
Timeline of events and treatment of Patient 1.

An 85‐year‐old man underwent transurethral resection of a bladder tumor for bladder cancer in X‐3, followed by right laparoscopic radical nephroureterectomy in X‐2 for recurrent right ureteral cancer. The pathological diagnosis was pure small cell carcinoma (pT3) with positive surgical margins; however, the patient did not consent to adjuvant therapy. Three months postoperatively, local recurrence on the dorsal surface of the bladder, peritoneal dissemination, and mesenteric lymph node enlargement were observed, along with renal dysfunction progression. After hemodialysis induction, the patient received six cycles of cisplatin (30 mg/m^2^) and irinotecan (30 mg/m^2^), followed by six cycles of pembrolizumab (200 mg/body) every 3 weeks. However, medication was withdrawn due to adrenal insufficiency caused by immune‐related AEs. Follow‐up CT 3 months post‐medication withdrawal showed increased peritoneal dissemination, prompting administration of EV. After two cycles of EV (1.25 mg/kg), PRs of the two peritoneal dissemination lesions were observed on CT based on the RECIST. However, disease progression was observed after four cycles (Fig. 1), prompting a shift to BSC. The AEs included grade‐3 neutropenia, grade‐2 skin rashes, and dysgeusia (Fig. 2). Neutropenia was managed with granulocyte colony‐stimulating factor, the rash with 0.3% heparinoid cream and oral fexofenadine (60 mg), while dysgeusia resolved spontaneously.

### Patient 2

**Fig. 3:**
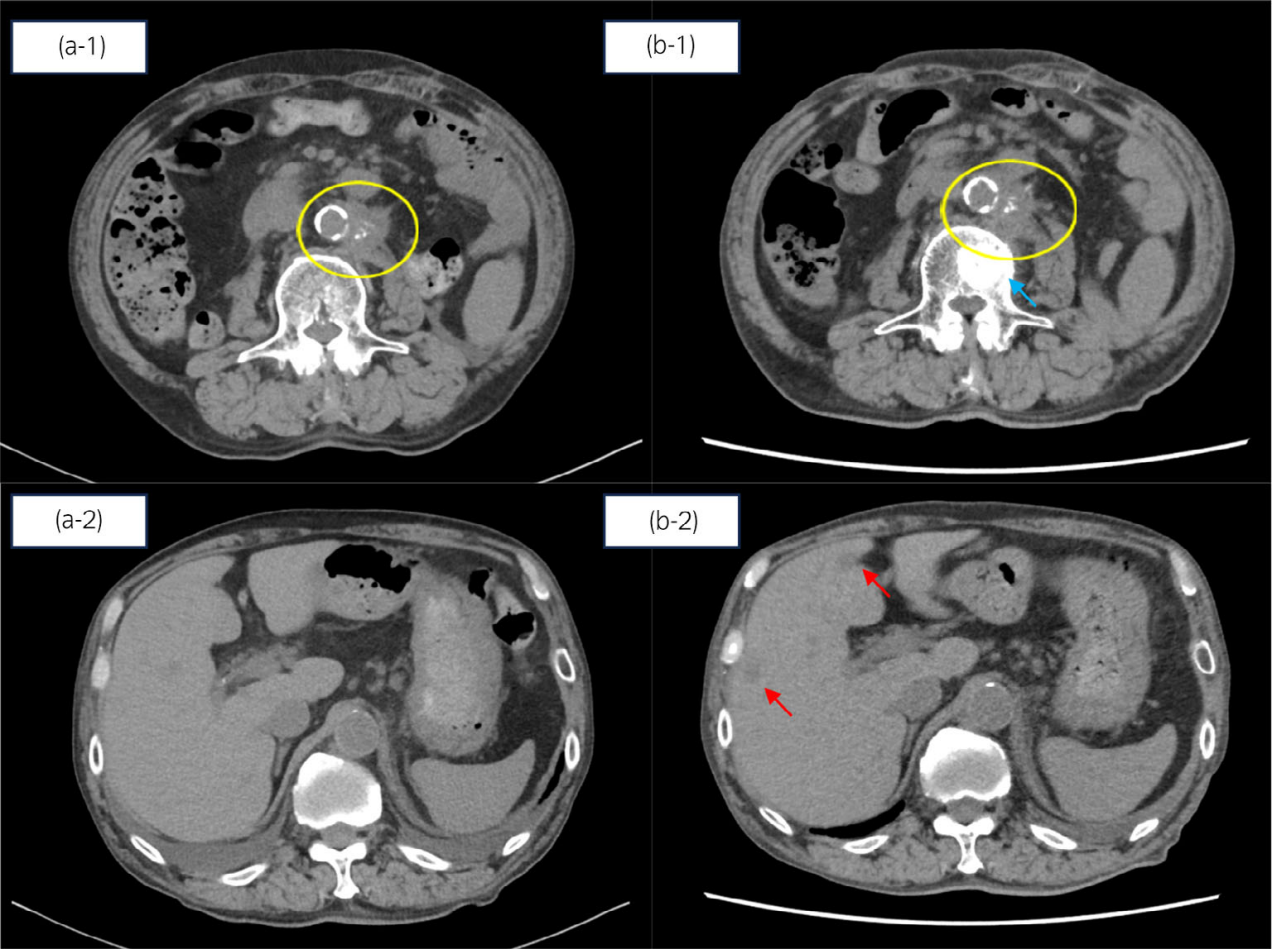
CT findings of Patient 2. CT from December Y‐1 before EV treatment demonstrating increase in size of para‐aortic lymph node (yellow circle, a‐1), normal lumbar vertebrae (a‐1), and normal liver (a‐2), and from March Y after two cycles of EV demonstrating progressive disease (PD), which is an increase in the size of para‐aortic lymph nodes (yellow circle, b‐1) and metastasis that appeared in the lumbar vertebrae (blue arrow, b‐1) and liver (red arrow, b‐2).

**Fig. 4:**
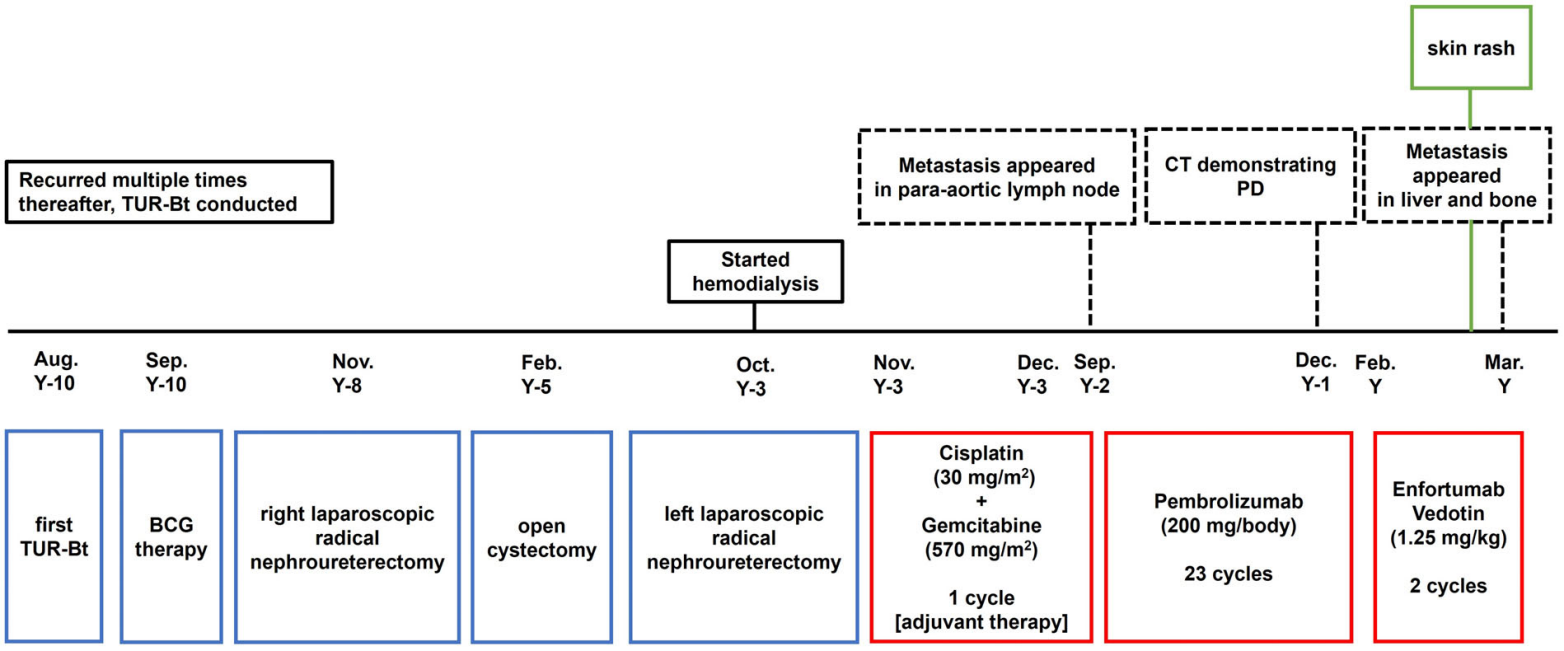
Timeline of events and treatment of Patient 2.

A 73‐year‐old man developed recurrent bladder cancer. He underwent right laparoscopic radical nephroureterectomy at Y‐8, radical cystectomy at Y‐5, and left laparoscopic radical nephroureterectomy at Y‐3, followed by hemodialysis. The pathological diagnosis was urothelial carcinoma (pT4), and the patient received adjuvant therapy consisting of gemcitabine (570 mg/m^2^) and cisplatin (30 mg/m^2^). However, treatment was halted after one cycle due to grade‐4 pancytopenia. Nine months post‐drug withdrawal, para‐aortic lymph node metastasis was detected, leading to treatment with pembrolizumab (200 mg/body) every 3 weeks. Although initially effective, the patient developed a para‐aortic lymph node metastasis after 23 cycles. Therefore, two cycles of EV (1.25 mg/kg) were administered starting. After two cycles, an increase in the existing lymph node metastases, along with new liver and bone metastases were observed (Fig. 3). PD was identified based on the RECIST, and the treatment approach was shifted to BSC. AEs included grade‐2 skin rash and pruritus (Fig. 4), relieved with hydrocortisone butyrate 1 mg (0.1%).

## Discussion

Treatment of mUC with medication has not yet been satisfactory. The overall response rate to platinum‐based chemotherapy and pembrolizumab, an ICI, for mUC is approximately 50% and 20%, respectively.^3^, ^4^ EV is a novel third‐line therapy for mUC, achieving a 40–44% overall response rate among patients with progressive disease during or after second‐line treatment.^1^, ^2^ This treatment option improves the prognosis of patients with mUC.

In this report, both patients with mUC showed disease progression after platinum‐based chemotherapy and ICI, making them eligible for EV treatment. However, these patients required hemodialysis due to ESRD. Although the impact of hemodialysis on EV pharmacokinetics has not been studied, BV, a similar antibody‐drug conjugate, exhibits roughly equivalent pharmacokinetics.^5^ Nanni *et al*. reported that BV was safely and effectively administered to hemodialysis patients with undifferentiated large‐cell lymphoma by reducing doses and ensuring a 24‐h interval between drug administration and hemodialysis initiation.^6^ However, a population pharmacokinetic analysis from the EV‐101 study indicated that EV exposure did not increase in patients with ESRD, thus not requiring dose adjustments based on renal function.^7^ Therefore, in our two cases, drug administration followed Nanni *et al*.'s BV protocol,^6^ maintaining at least a 24‐h interval between administration and hemodialysis initiation, with the full dose administered. One patient achieved a PR, whereas the other experienced PD. Eventually, in both cases, the disease progressed. There is no established post‐EV treatment for UTUC, not only in dialysis patients, and no further treatment was administered due to the patient's advanced age and potential decline in quality of life from additional invasive treatments. Regarding safety, neutropenia was the only AE of grade‐3 or higher. In both cases, a mild skin rash developed. While skin rash is a common AE of EV, it can also emerge up to a year post‐treatment with pembrolizumab, an EV pre‐treatment drug.^8^ Identifying the exact causative drug in these instances was challenging, but rash onset might be delayed due to ICI effects even after early EV discontinuation.

The 5‐year metastasis‐free survival rate after total nephroureterectomy for UTUC is 72–82%.^9^ Furthermore, the 5‐year recurrence‐free survival rate after complete urinary tract extirpation is 67%.^10^ Therefore, the number of recurrent or metastatic UTUC cases postoperatively is relatively high. Cisplatin, the primary platinum drug used as first‐line chemotherapy for mUC, frequently causes renal dysfunction.^11^ It is assumed that patients with mUC, who have progressed to the point where EV administration is considered, include those who have to undergo hemodialysis because of worsening renal function after these multidisciplinary treatments, such as surgery and chemotherapy. However, this report and the one by Isoda *et al*.,^12^ are the only to describe the application of EV in patients undergoing hemodialysis, with the latter describing a complete response and no grade‐3 or higher AEs.^12^ Thus, EV may be an effective and safe treatment option for patients undergoing hemodialysis. In our report, the first patient was diagnosed with pure small cell carcinoma, a non‐urothelial type with low nectin‐4 expression, significantly reducing EV's effectiveness.^13^, ^14^ Despite the expected reduced effectiveness, the treatment was successful in this patient; however, the specific reasons remain unclear. Further research is needed on nectin‐4 expression and EV's impact on non‐urothelial subtypes, including small cell carcinomas.

## Conclusion

EV has been successfully administered at full dose to mUC patients with ESRD on hemodialysis.

## Author contributions

Shintaro Mori: Writing – original draft; writing – review and editing. Tomohiro Matsuo: Writing – original draft; writing – review and editing. Hiroyuki Honda: Writing – original draft; writing – review and editing. Kyohei Araki: Writing – original draft; writing – review and editing. Kensuke Mitsunari: Writing – original draft; writing – review and editing. Kojiro Ohba: Writing – original draft; writing – review and editing. Yasushi Mochizuki: Writing – original draft; writing – review and editing. Ryoichi Imamura: Supervision; writing – original draft; writing – review and editing.

## Conflict of interest

The authors declare no conflict of interest.

## Approval of the research protocol by an Institutional Reviewer Board

No ethical approval was required for this case report.

## Informed consent

Written informed consent was obtained from the patients.

## Registry and the Registration No. of the study/trial

Not applicable.
